# Association of Geriatric Emergency Department Care With Hospitalization and Mortality in Older Adults

**DOI:** 10.1111/jgs.70421

**Published:** 2026-04-05

**Authors:** Yuting Qian, Cameron Gettel, Jasmine Su, Elyssa F. L. Grogan, Inessa Cohen, Craig Rothenberg, Xi Chen, Ula Hwang

**Affiliations:** 1 Department of Health Policy and Management, Yale University, New Haven, Connecticut, USA; 2 Department of Emergency Medicine, Yale University School of Medicine, New Haven, Connecticut, USA; 3 Department of Emergency Medicine, NYU Grossman School of Medicine, New York, New York, USA; 4 Program of Computational Biology and Bioinformatics, Yale University School of Medicine, New Haven, Connecticut, USA; 5 Department of Economics, Yale University, New Haven, Connecticut, USA; 6 National Bureau of Economic Research, Cambridge, Massachusetts, USA; 7 Department of Population Health, NYU Grossman School of Medicine, New York, New York, USA; 8 Geriatrics Research, Education and Clinical Center, James J. Peters VA Medical Center, Bronx, New York, USA

**Keywords:** emergency department care, geriatric emergency department accreditation, hospital admission, mortality

## Abstract

**Background:**

Since 2018, the Geriatric Emergency Department (GED) Accreditation Program has recognized Emergency Departments (EDs) that provide high-quality care tailored to older adults. GEDs have expanded rapidly across the United States in recent years, but little is known about how GED care is associated with patient outcomes, including hospital admissions and subsequent mortality.

**Methods:**

We used the 2018–2021 Health and Retirement Study (HRS)-Medicare linked data of adults aged ≥ 65 years. We supplemented these data with the American College of Emergency Physicians (ACEP) GED accreditation list and American Hospital Association (AHA) data. Receipt of acute care in a GED was defined as having an ED visit at a GED. Patient-level analyses were conducted using each individual’s most recent ED visit. Multivariable logistic regression models were used to estimate associations between receipt of acute care in a GED and outcomes of hospital admission and 30-day mortality, adjusting for patient demographics, socioeconomic status, health conditions, ED visit severity, and hospital-level characteristics.

**Results:**

Among 4563 older adults who had an ED visit, 270 (5.9%) received acute care in GEDs and 4293 (94.1%) received non-GED care. Compared with those treated in non-GEDs, patients treated in GEDs had significantly lower odds of hospital admission (OR, 0.61; 95% CI, 0.42–0.87; *p* < 0.01) and 30-day mortality (OR, 0.62; 95% CI, 0.40–0.96; *p* < 0.05). Subgroup analyses showed that the association with admission was more pronounced among adults aged 65–80 years (OR, 0.43; 95% CI, 0.24–0.76; *p* < 0.01) and non-Hispanic White individuals (OR, 0.51; 95% CI, 0.34–0.78). An association with lower mortality was observed among non-Hispanic White individuals (OR, 0.51; 95% CI, 0.30–0.87; *p* < 0.05).

**Conclusions:**

GED care was associated with lower odds of hospital admissions and 30-day mortality among older adults. Broader implementation may expand the reach of GED programs across diverse populations.

## 1 ∣ Introduction

The emergency department (ED) provides critical medical care to older adults, who visit more frequently than younger patients and have almost double the proportion of ED encounters nationally [1, 2]. Older ED patients often present with complex needs and have increased risk of multimorbidity, polypharmacy, dementia, delirium, and falls, which complicate ED decision-making and increase hospitalization risk [3-8]. The ED decision to admit an older adult is consequential, as admissions are costly and may expose patients to health care-associated infections [9], iatrogenic complications, functional decline, and subsequent mortality [10, 11]. As the population ages, the challenge of delivering high-quality and safe ED care for older adults will become increasingly urgent.

In recognition of the unique medical needs of older adults, the Geriatric Emergency Department (GED) was conceptualized two decades ago addressing gaps in the delivery of acute care for this population [12]. In 2014, the first GED guidelines were published and approved by the American College of Emergency Physicians (ACEP), the Society for Academic Emergency Medicine, the American Geriatrics Society, and the Emergency Nurses Association [13]. In 2018, ACEP launched its Geriatric Emergency Department Accreditation (GEDA) Program, recognizing EDs delivering targeted geriatric care [14]. Accredited GEDs attest to implementing geriatric practices and tracking outcome measures aligned with the GED guidelines in multiple domains, including staffing, care processes, care transitions, and enhancements to the physical environment. GEDA criteria increase in stringency from Level 3 (Bronze) to Level 2 (Silver), to Level 1 (Gold), with greater requirements across these domains and higher costs [14]. As of December 2021, 284 EDs were accredited in the United States (US).

The rapid expansion of GEDs underscores the need for a comprehensive evaluation of patient-centered outcomes associated with GED care. Previous research on GEDs has found mixed evidence for the role of GED initiatives in outcomes such as hospital admissions, ED revisit rates, geriatric consultations, and healthcare cost-savings [15-21]. Many of these studies were single-site, focused on specific interventions (e.g., transitional care nurses or geriatric syndrome screening), and were limited in generalizability to GED care. Although one nationwide study evaluated process outcomes, such as diagnosis of geriatric syndromes, ED length of stay, and revisit rates [22], hospital admissions remain understudied, and the association between GED care and mortality has not been evaluated.

To address these research gaps, our study leveraged nationally representative, longitudinal, patient- and encounter-level data to examine the association between receiving acute care in GEDs and two patient-centered outcomes: hospital admissions and mortality. We assessed the heterogeneity in these associations across patient subgroups.

## 2 ∣ Methods

### 2.1 ∣ Data and Sample

We used data from the 2018–2021 Health and Retirement Study (HRS) linked with fee-for-service (FFS) Medicare claims. The HRS is a nationally representative biennial survey of US adults with detailed demographic, socioeconomic, and health care utilization data through linked Medicare claims [23]. Our analytic sample included HRS respondents aged 65 years or older enrolled in Medicare FFS with at least one ED visit between 2018 and 2021. To identify EDs that implemented GED practices, we relied on the ACEP accreditation list [14], which included ED site names and the application initiation date for GED accreditation. To incorporate hospital-level characteristics, we linked the ACEP list of GEDs to the 2018–2021 American Hospital Association (AHA) database by fuzzy-matching facility and city names, a string similarity-based matching method commonly used in prior literature (Methods S1) [24-26]. The HRS-Medicare claims data were then linked to the AHA database and the ACEP accreditation list using CMS Certification Numbers (CCNs).

The primary exposure was receipt of acute care in a GED (hereafter referred to as “GED care”), defined as visiting a GED during the study period. The earliest application date was used to designate GED status, such that an ED was considered a GED once it had applied for accreditation. This decision was made because sites began implementing GED practices before formal accreditation. Patients with at least one GED visit were classified as receiving GED care, and those without GED visits were classified as receiving non-GED care.

**FIGURE 1 ∣:**
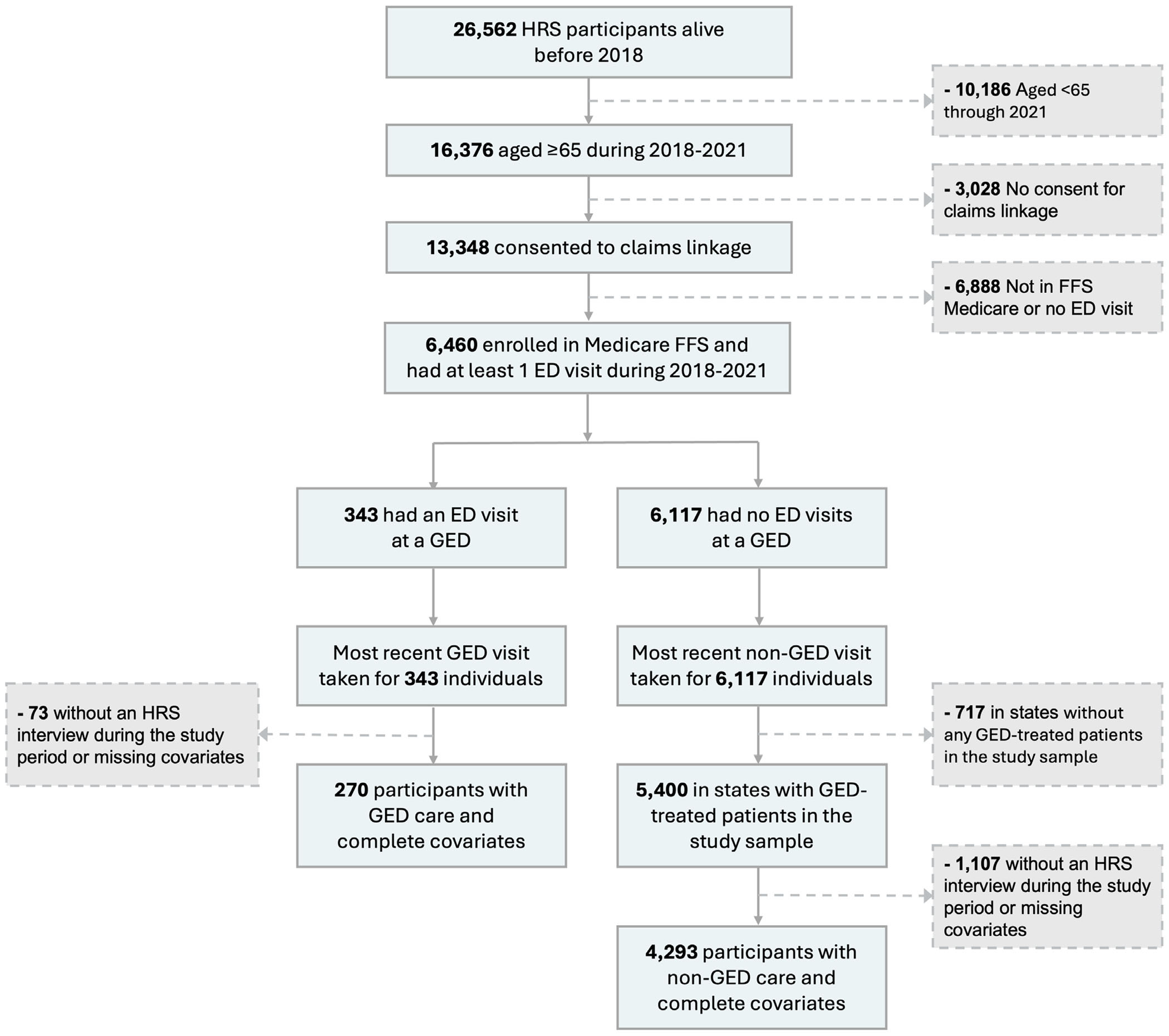
Generation of the analytic sample. HRS, Health and Retirement Study; ED, emergency department; GED, Geriatric Emergency Department; FFS, fee-for-service. *Note:* The study sample included HRS respondents aged 65 years or older who consented to Medicare records linkage, were enrolled in Medicare FFS, and had at least one ED visit between 2018 and 2021. Individuals were classified as having received GED care if they had ED visits at a GED during the study period, and those with no GED visits were classified as the control group. The analytic sample was constructed at the patient level using each patient’s most recent ED visit, defined as the most recent GED visit for those who received GED care (treated group) and the most recent ED visit for those who received only non-GED care (control group). To improve comparability, the control group was restricted to patients residing in states represented in the treated group. Individuals without an HRS interview (and thus with certain covariates unavailable) or with otherwise missing covariates in the year of their most recent ED visit were excluded.

The analytic sample was constructed at the patient level, indexing each patient’s most recent ED visit, defined as the latest GED visit for those who received GED care (treated group) and the most recent ED visit for those who received only non-GED care (control group). We used the most recent rather than the initial visit to reflect periods when GED practices were more fully implemented. To improve comparability, the control group was restricted to patients residing in states represented in the treated group. Individuals with missing covariate data were excluded. Figure 1 presents a flowchart of the sample selection process. The Yale Institutional Review Board approved this study with a waiver of informed consent as the study used secondary data and posed no more than minimal risk. This study followed the Strengthening the Reporting of Observational Studies in Epidemiology (STROBE) reporting guidelines [27].

### 2.2 ∣ Outcome Measures

The primary outcome was hospital admission from the ED, defined as inpatient admission or placement in observation status within 2 days of the index ED visit. As in prior studies [28, 29], observation stays (identified using revenue center code 0763) [30] were considered clinically equivalent to admissions because patients continued to receive care rather than being discharged home. The secondary outcome was mortality, defined as death within 30 days of the ED visit. This window allowed us to capture deaths shortly after the ED visit as well as after other events including hospitalization, length of stay, care transitions, and early readmission [31, 32]. Date of death came from the Medicare Beneficiary Summary File (MBSF).

### 2.3 ∣ Covariates

Covariates were selected based on characteristics likely to be associated with the outcomes of hospital admission and mortality as well as with GED care. These included individual-level demographics (age, sex, race and ethnicity), socioeconomic status (education, partner status, and dual eligibility), health status (number of chronic conditions), ED visit severity, and hospital-level characteristics (teaching hospital status, hospital rurality, and staffed hospital beds). HRS data contained age, sex, race and ethnicity, education, and partner status. Because the HRS is a biennial survey, time-varying covariates available only in the HRS (e.g., partner status) were derived annually by matching each patient’s most recent ED visit to the closest interview year (i.e., values for 2019 and 2021 were taken from the 2020 interview). Dual eligibility, used as a proxy for social risk in prior studies [33, 34], was obtained from the MBSF and defined as receipt of full or partial Medicaid benefits in any month of a given calendar year. The Chronic Conditions Data Warehouse (CCW) was used to construct the number of chronic conditions for each patient.

To measure the severity of ED visits, we used the updated version of the New York University ED Algorithm to classify each visit based on the primary diagnosis [35]. The algorithm assigns probabilities that the ED visit was emergent, not preventable; emergent, preventable; emergent, primary care treatable; nonemergent; or related to injury, mental health, alcohol, or drug use, or unclassifiable. Following prior literature [36, 37], we classified a visit as emergent if the sum of the probabilities of “emergent, not preventable” and “emergent, preventable” exceeded 50%, and as nonemergent if the sum of the probabilities of “nonemergent” and “emergent, primary care treatable” exceeded 50%. All remaining visits were grouped into a single “other” category because of small cell sizes in categories other than injury and unclassifiable.

### 2.4 ∣ Statistical Analysis

We conducted patient-level analyses using multivariable logistic regression testing if treatment in a GED was associated with hospital admission and 30-day mortality, controlling for all covariates described above. We also included an indicator for the pre-coronavirus disease (COVID-19) period (before March 2020) versus the COVID-19 period (March 2020 to 2021) and indicators for quarters to account for patient-invariant and unobserved temporal differences. All standard errors were estimated using Huber-White estimators of variance. All regression analyses incorporated HRS sampling weights to adjust for unequal probability of inclusion in the sample and to produce nationally representative estimates. In secondary analyses, we repeated the main analyses within subgroups stratified by age (> 80 vs. ≤ 80 years) and by race and ethnicity (non-Hispanic White vs. non-White) and tested for differences by including interaction terms between each subgroup indicator and the treatment variable in the regression models.

We tested the robustness of our findings. First, we applied alternative definitions of hospital admission, including inpatient admission within 1 and 2 days of the ED visit, excluding observation status. Second, we conducted a sensitivity analysis excluding patients potentially transferred to or from another ED. Third, we repeated the analyses accounting for freestanding EDs. Fourth, we performed analyses separately for the pre-COVID-19 and COVID-19 periods, as the pandemic may have altered ED utilization and admission practices. Fifth, to examine differences in early mortality, we studied 7-day mortality as an alternative outcome. Finally, we conducted a placebo test to assess whether findings were driven by unobserved pre-existing hospital-level differences rather than GED implementation. We looked at “pseudo-GED visits” defined as patients’ first ED visit to sites that later became GEDs but occurred prior to GEDA application, and compared outcomes with first ED visits among patients who never visited a GED site during the study period, adjusting for the same covariates as in the main analysis. Since no site had implemented GED practices at that time, any observed differences would reflect pre-existing hospital-level differences rather than GED care.

In the main results, we present estimates from logistic regression as odds ratios (ORs) and report absolute differences based on marginal effects. In Supporting Information, we report risk ratios using Poisson regression with robust variance estimators. The study was conducted using Stata statistical software version 14.1 (StataCorp). Two-sided *p* < 0.05 was considered statistically significant.

## 3 ∣ Results

A total of 6460 HRS respondents aged 65 years or older had at least one ED visit in Medicare FFS claims. After additional sample restrictions (Figure 1), the analytic sample included 4563 older adults with an ED visit, representing a weighted total of 25,317,444 adults nationally. Among them, 270 patients (5.9%) received care in a GED, and 4293 (94.1%) received care in a non-GED. Sample characteristics by GED care status are shown in Table 1. Patients who received and did not receive GED care were similar in age, sex, education, partner status, dual eligibility, health conditions, and ED visit severity. However, compared with those receiving non-GED care, patients receiving GED care were less likely to be non-Hispanic White (64.8% vs. 68.7%; *p* = 0.061) or have their ED visit at rural hospitals (6.3% vs. 13.9%; *p* < 0.001), and more likely to visit teaching hospitals (83.4% vs. 74.1%; *p* < 0.001) and hospitals with more than 500 beds (38.1% vs. 24.4%; *p* < 0.001). Weighted sample characteristics showed similar patterns (Table S1).

**FIGURE 2 ∣:**
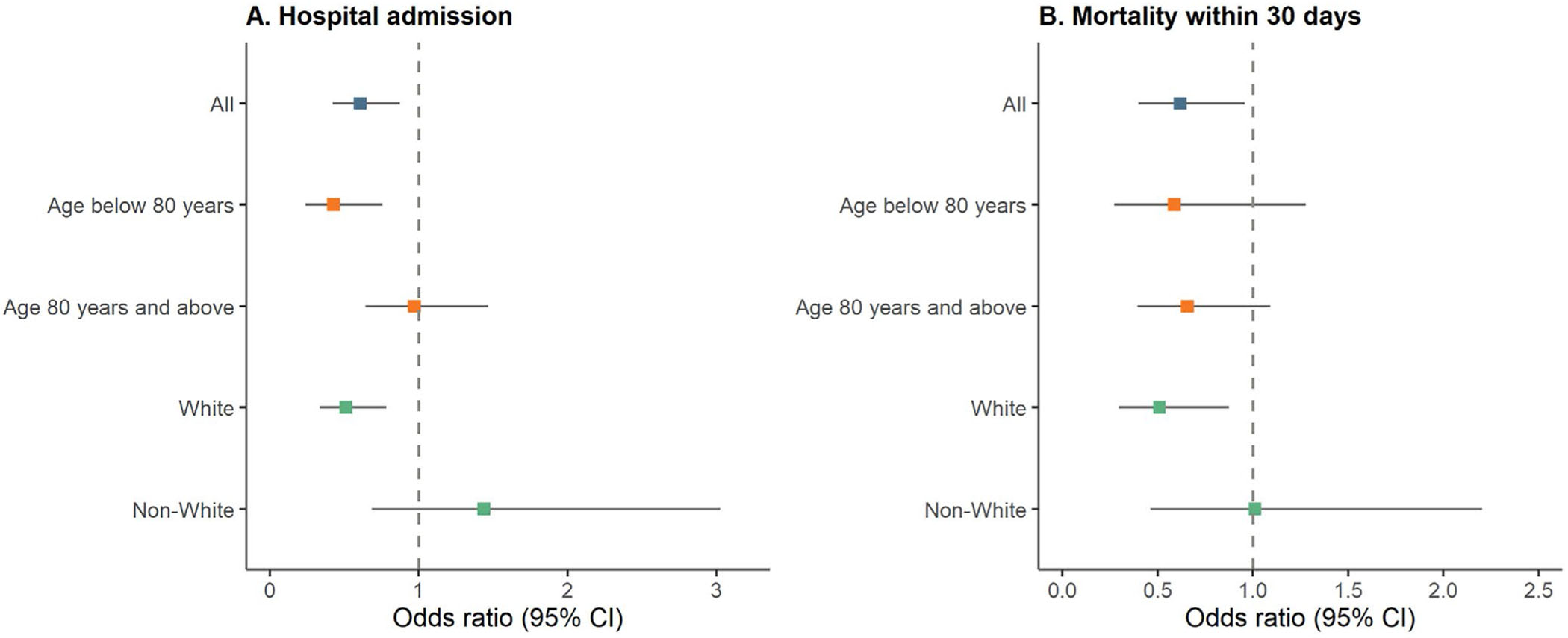
Adjusted association between receipt of Geriatric Emergency Departments (GED) care and patient outcomes following an Emergency Department visit. *Note:* The figure shows adjusted associations between receipt of GED care and patient outcomes, including hospital admission and 30-day mortality from the date of the ED visit, for the overall population and for subgroups stratified by age (< 80 vs. ≥ 80 years) and race/ethnicity (non-Hispanic White vs. other). Squares represent odds ratios, and error bars indicate 95% CIs. The referent group is patients who received non-GED care. Details of the regression models are provided in Section 2 of the manuscript.

In unadjusted analyses, 63.3% of patients treated in GEDs and 65.5% treated in non-GEDs were admitted after the ED visit; 30-day mortality was 13.7% and 18.5%, respectively (Table S2). After adjusting for covariates in multivariable logistic regression, patients who received care in GEDs had significantly lower odds of hospital admission (OR, 0.61 [95% CI, 0.42–0.87]; absolute difference, −9.71 [95% CI, −16.80 to −2.63] percentage points; *p* < 0.01) and 30-day mortality (OR, 0.62 [95% CI, 0.40–0.96]; absolute difference, −6.11 [95% CI, −11.69 to −0.52] percentage points; *p* < 0.05) compared with those who received non-GED care (Figure 2, Table 2).

In secondary analyses, among patients younger than 80 years, GED care was associated with lower odds of hospital admission (OR, 0.43 [95% CI, 0.24–0.76]; absolute difference, −16.51 [95% CI, −27.49 to −5.53] percentage points; *p* < 0.01) compared with patients who received non-GED care (Figure 2, Table 2). No significant association was observed among patients aged 80 years or older (OR, 0.97; 95% CI, 0.64–1.47). Among non-Hispanic White patients, GED care was associated with lower odds of hospital admission (OR, 0.51 [95% CI, 0.34–0.78]; absolute difference, −13.47 [95% CI, −21.94 to −5.00] percentage points; *p* < 0.01) and 30-day mortality (OR, 0.51 [95% CI, 0.30–0.87]; absolute difference, −8.35 [95% CI, −15.08 to −1.61] percentage points; *p* < 0.05) compared with non-GED care, whereas no significant associations were observed among non-White patients for either outcome (admission: OR, 1.44; 95% CI, 0.68–3.03; mortality: OR, 1.01; 95% CI, 0.46–2.20). The association between GED care and hospital admission also differed significantly across age, race and ethnicity subgroups (Table S3).

Our results were robust to sensitivity analyses using alternative definitions of hospital admission, with GED care associated with lower odds of admission when excluding observation stays (OR, 0.55; 95% CI, 0.39–0.78; *p* < 0.01) and when restricting to admission within 1 day excluding observation stays (OR, 0.59; 95% CI, 0.42–0.83; *p* < 0.01) (Table 3). Results were also consistent when excluding patients potentially transferred to or from another ED (Table S4), when additionally adjusting for freestanding EDs (Table S5), and when restricting the analysis to the pre-COVID-19 period (Table S6). We did not observe a statistically significant association between GED care and 7-day mortality (OR, 0.73; 95% CI, 0.38–1.39) (Table S7), which may reflect that associations with mortality are driven by downstream care decisions and processes over a relatively longer time horizon. Additionally, results from the placebo test showed that GED care during the pre-GED period was not associated with lower odds of hospital admission (OR, 1.01; 95% CI, 0.73–1.39) or 30-day mortality (OR, 0.80; 95% CI, 0.41–1.56) (Table 3), suggesting the associations observed in the analysis are unlikely to be driven solely by unobserved preexisting hospital characteristics. Finally, Poisson regression estimates were consistent with the main findings (Table S8).

## 4 ∣ Discussion

In this retrospective cohort study using a sample of older adults with US ED visits between 2018 and 2021, we provide the first nationally representative evidence of the association between GED care and patients’ hospital admission from the ED and their subsequent mortality. We found that GED care was associated with significantly lower odds of hospital admission and 30-day mortality following an ED visit. Moreover, these associations were most pronounced among non-Hispanic White patients, with no significant associations observed among non-White patients. Lower odds of hospital admission were also observed among patients younger than 80 years, but not those aged 80 years or older.

Prior studies have suggested potential benefits of innovative ED programs for older adults, such as the Geriatric Emergency Department Innovations in Care Through Workforce, Informatics, and Structural Enhancements (GEDI WISE), which have been associated with lower admission rates, fewer future ED visits, reduced 30-day readmissions, and cost savings [19-21]. Evaluations of GED-accredited sites have also reported improved process outcomes, including higher recognition of geriatric syndromes and shorter ED lengths of stay [22]. Our study expands this literature with national evidence on the association between GED care and lower odds of hospital admission and 30-day mortality following an ED visit. Furthermore, we found these associations were not evenly distributed across patient subpopulations.

Several mechanisms may explain the observed associations between GED care and lower odds of hospital admissions and short-term mortality. GEDs incorporate age-friendly environments, ED staff with geriatric training, and structured care processes that emphasize geriatric assessment and coordination. EM staff with geriatric education and dedicated roles (e.g., transitional care nurses) in GEDs may enable more comprehensive evaluation of older adults and closer monitoring. Mechanistically, structured geriatric care processes may facilitate earlier identification and management of geriatric syndromes [38], reduce exposure to harmful interventions (e.g., urinary catheter minimization), and enhance care transitions [22, 39, 40], which altogether may reduce complications, prevent unnecessary admissions, and support safer discharge planning.

Our findings that GED care was associated with lower odds of admissions and mortality among non-Hispanic White but not non-White patients suggest that structural and contextual factors beyond clinical need may shape its effectiveness. Prior studies indicate that health system factors such as limited outpatient follow-up, inadequate access to specialty or primary care, and lack of social supports may influence physicians’ admission decisions [41, 42]. These barriers are more prevalent among Black and Hispanic older adults, who may face structural disadvantages in accessing timely and continuous care [42, 43]. Limited access to care, resources, and follow-up engagement among minoritized populations may also attenuate the association between GED care and mortality. Provider- and system-level biases in care may also contribute. Prior evidence shows Black patients are less likely to have symptoms recognized or receive indicated therapies and often face longer wait times than White patients [44, 45]. Together, these disparities underscore the importance of pairing GED innovations with broader efforts to increase equitable outpatient access, support continuity of care, and reduce biases in care provision across diverse patient populations. At the same time, given prior evidence of inconsistent reporting in the geriatric emergency medicine (GEM) literature [46], efforts to reduce these disparities will require transparent research that reports analyses stratified by race and ethnicity, gender and sex, primary language, rurality, and other relevant factors.

We found that GED care was associated with lower odds of admissions among patients younger than 80 years, whereas such an association was not observed among those 80 years and older. This difference likely reflects the greater medical complexity, functional limitations, and frailty of the oldest age group, who have higher admission rates and for whom hospitalization decisions are more often acuity-driven and less discretionary [42, 47]. In contrast, admissions among younger older adults may be more sensitive to interventions targeting geriatric ED patients, enabling GED care models to help avert hospitalization and support safer discharge.

Taken together, our findings highlight the value of GED care in improving patient outcomes and underscore the role of the GEDA program as a framework recognizing EDs delivering such care. Pursuing GEDA requires investment, including application fees (ranging from $5000 to $15,000 to apply for Bronze to Gold levels), staff training, and equipment. In addition, the GEDA program is a tiered system, in which Bronze, Silver, and Gold levels represent increased commitment to GED staffing and care process implementation, likely entailing higher overall costs. Prior work suggests these upfront costs associated with GED care may be justified by revenue generation and improvements in patient safety [18]. Although our study focused on overall GED care rather than differences across accreditation levels, it is possible that higher accreditation is associated with greater improvements in patient outcomes and economic return on investment.

### 4.1 ∣ Limitations

We acknowledge several limitations in our study. First, our sample may not generalize to Medicare Advantage enrollees or to adults under age 65. Second, our analysis was limited to EDs with CCNs and may not generalize to federally operated facilities, such as Veterans Affairs hospitals, for which CCNs were not available. Third, a small proportion of GEDs with missing CCN identifiers may have been misclassified as control sites, which would likely bias our estimates toward the null. Fourth, the relatively small number of patients who received GED care likely limited the statistical power of the subgroup analyses. Fifth, we defined GED status using the application date rather than the later accreditation date, which would lead to an underestimation of the association. However, this approach was carefully considered, given that GED implementation naturally precedes accreditation. In addition, because we did not observe whether individual patients received specific GED services, incomplete or imperfect implementation of GED practices would likely attenuate the estimated associations and bias the results toward the null. Sixth, because our study is observational, we could not rule out residual confounding from unobserved patient- or provider-level factors not captured in our data, such as ED size and crowding at the time of presentation, or the availability of community and informal care. Although we adjusted for a rich set of covariates, our findings should be interpreted as associations rather than causal effects. Our placebo tests, however, suggest that the observed associations between patient outcomes and GED care are unlikely to be driven solely by preexisting hospital characteristics. While randomized trials of GED versus non-GED care would provide the strongest causal evidence, they may be impractical or ethically challenging. Future studies with quasi-experimental designs [48], larger samples, longer follow-up, and plausibly exogenous variation [49] in GED adoption would strengthen causal inference and examine a broader range of process and clinical outcomes. Finally, because of the small number of Silver- and Gold-level GEDA sites and the lack of detailed information on accreditation timing at the time of the ED visit, we could not reliably assess heterogeneity by GEDA level. Future studies with more detailed accreditation data and larger samples on Silver- and Gold-level sites could evaluate differences across accreditation levels.

## 5 ∣ Conclusions

Our study provides national evidence on the association between GED care and lower odds of hospital admissions and mortality, highlighting the relevance of this model to acute care delivery for older adults. Although the number of accredited GED sites has been increasing (612 EDs accredited as of early 2026 in the US), the proportion of older adults receiving care in these settings remains small. Policy initiatives can further incentivize and expand the reach of GED care. Our findings also indicate that the association between GED care and hospital admission varies across populations, with differences in effectiveness likely driven by variability in access to care, availability of social supports outside the ED, and biases in care provision. Ensuring that expansion of GED care is combined with efforts to address these inequities is pivotal to equitable outcomes across diverse populations.

## Supplementary Material

Supplementary Tables

Additional supporting information can be found online in the Supporting Information section. **Table S1:** Weighted characteristics of health and retirement study participants receiving care in Geriatric Emergency Departments (GEDs) versus non-GEDs. **Table S2:** Unadjusted outcomes among health and retirement study participants receiving care in Geriatric Emergency Departments (GEDs) versus non-GEDs. **Table S3:** Heterogeneity of the association between receipt of geriatric emergency department care and outcomes by age and race/ethnicity. **Table S4:** Sensitivity analysis excluding patients transferred to or from another ED. **Table S5:** Sensitivity analysis adjusting for freestanding emergency departments. **Table S6:** Association of geriatric emergency department care with patient outcomes before and during the COVID-19 pandemic. **Table S7:** Adjusted association between receipt of geriatric emergency department care and 7-day mortality following an emergency department visit. **Table S8:** Poisson regression estimates for the association between receipt of geriatric emergency department care and patient outcomes following an emergency department visit. **Methods S1**: Fuzzy matching procedure for data linkage.

## Figures and Tables

**TABLE 1 ∣:** Characteristics of health and retirement study participants receiving care in Geriatric Emergency Departments (GEDs) versus non-GEDs.

	No. (%)			
Characteristics	Care in GED^a^	Care in non-GED^a^	Full sample	*p*
Number of patients, unweighted	270 (5.9)	4293 (94.1)	4563	
Number of patients, weighted^b^	1,574,617 (6.2)	23,742,827 (93.8)	25,317,444	
Patient characteristics				
Age, years old				
65–69	35 (13.0)	609 (14.2)	644 (14.1)	0.290
70–74	44 (16.3)	643 (15.0)	687 (15.1)	
75–79	36 (13.3)	771 (18.0)	807 (17.7)	
80–84	69 (25.6)	957 (22.3)	1026 (22.5)	
≥ 85	86 (31.9)	1313 (30.6)	1399 (30.7)	
Male	105 (38.9)	1708 (39.8)	1813 (39.7)	0.770
Race and ethnicity				
Non-Hispanic White	175 (64.8)	2951 (68.7)	3126 (68.5)	0.061
Non-Hispanic Black	57 (21.1)	744 (17.3)	801 (17.6)	
Hispanic	27 (10.0)	505 (11.8)	532 (11.7)	
Non-Hispanic Other	11 (4.1)	93 (2.2)	104 (2.3)	
High school graduate or higher	202 (74.8)	3148 (73.3)	3350 (73.4)	0.592
With partner	125 (46.3)	2021 (47.1)	2146 (47.0)	0.803
Dual-eligible	58 (21.5)	1006 (23.4)	1064 (23.3)	0.462
Number of chronic conditions^c^, mean (SD)	3.07 (1.44)	3.13 (1.57)	3.13 (1.56)	0.499
ED visit severity				
Emergent	56 (20.7)	982 (22.9)	1038 (22.7)	0.317
Nonemergent	50 (18.5)	905 (21.1)	955 (20.9)	
Others^d^	164 (60.7)	2406 (56.0)	2570 (56.3)	
Hospital-level characteristics^e^				
Rural hospital	17 (6.3)	597 (13.9)	614 (13.5)	< 0.001
Teaching hospital				
Major teaching hospital	99 (36.7)	579 (13.5)	678 (14.9)	< 0.001
Minor teaching hospital	126 (46.7)	2603 (60.6)	2729 (59.8)	
Nonteaching hospital	45 (16.7)	1111 (25.9)	1156 (25.3)	
Total facility beds set up and staffed				
1–99	31 (11.5)	651 (15.2)	682 (14.9)	< 0.001
100–299	59 (21.9)	1527 (35.6)	1586 (34.8)	
300–499	77 (28.5)	1069 (24.9)	1146 (25.1)	
≥ 500	103 (38.1)	1046 (24.4)	1149 (25.2)	

Abbreviations: ED, Emergency Department; GED, Geriatric Emergency Department.

a The table compares characteristics of patients who received care in GEDs with those who only received care in non-GEDs. The sample included individuals with an ED visit between 2018 and 2020 and was constructed at the patient level using the most recent GED visit for patients who received GED care and the most recent ED visit for those who only received non-GED care.

b The number of weighted observations was calculated using Health and Retirement Study (HRS) analytic weights to represent US adults aged 65 years or older.

Weighted sample characteristics were presented in Table S1.

c Chronic conditions data were obtained from the Chronic Condition Warehouse (CCW) linked to the Health and Retirement Study (HRS) survey respondents.

Conditions were categorized in alignment with the HRS survey and included eight categories: high blood pressure, diabetes, cancer, lung disease, heart disease, stroke, arthritis, and psychiatric problems. HRS self-reported conditions were used to supplement CCW indicators when CCW data were unavailable. Missing self-reported conditions for a given year were imputed using the most recent nonmissing value within the prior 4-year window.

d The remaining category includes visits related to injury, mental health, alcohol or drug use, as well as visits that could not be classified.

e Hospital-level characteristics were derived from American Hospital Association data linked to Medicare claims and reflect the hospital of each patient’s most recent ED visit (the most recent GED visit for GED patients and the most recent ED visit for non-GED patients).

**TABLE 2 ∣:** Regression estimates for the association between receipt of Geriatric Emergency Department care and patient outcomes following an Emergency Department visit.

Samples	Hospital admission^a^		Death within 30 days of ED visit	
	OR^b^ (95% CI)	Absolute difference^c^, percentage points (95% CI)	OR^b^ (95% CI)	Absolute difference^c^, percentage points (95% CI)
Panel A. Overall sample	0.607**(0.421 to 0.874)	−9.71**(−16.80 to −2.63)	0.619*(0.400–0.958)	−6.11*(−11.69 to −0.52)
Observations	4520	4520	4563	4563
Panel B. Subgroups by age				
Age ≥ 80 years old	0.970(0.642 to 1.467)	−0.57(−8.39 to 7.24)	0.658(0.396–1.093)	−6.78(−15.00 to 1.44)
Observations	2400	2400	2425	2425
Age < 80 years old	0.427**(0.241 to 0.757)	−16.51**(−27.49 to −5.53)	0.589(0.272–1.278)	−5.26(−12.99 to 2.47)
Observations	2120	2120	2138	2138
Panel C. Subgroups by race/ethnicity				
White^d^	0.512**(0.335 to 0.782)	−13.47**(−21.94 to −5.00)	0.510*(0.297–0.874)	−8.35*(−15.08 to −1.61)
Observations	3104	3104	3126	3126
Non-White^d^	1.438(0.683 to 3.028)	5.89(−6.20 to 17.99)	1.011(0.464–2.203)	0.15(−10.59 to 10.89)
Observations	1416	1416	1437	1437

*Note:* Significance levels:

** *p* < 0.01,

* *p* < 0.05.

Abbreviations: ED, Emergency Department; GED, Geriatric Emergency Department.

a Patients who died in the outpatient ED before being admitted to an inpatient or observation stay were not at risk for admission and were therefore excluded from the hospital admission outcome.

b Odds ratios presented in this table correspond to Figure 2.

c Absolute differences were estimated from logistic regressions generating predictive margins and represent the change in predicted outcome probabilities between patients who received GED care and those who received non-GED care, adjusted for covariates.

d White subgroups refer to non-Hispanic White individuals, and non-White subgroups refer to other racial and ethnic groups, including non-Hispanic Black, Hispanic, and others.

**TABLE 3 ∣:** Sensitivity analyses with alternative outcome definition and placebo test.

Outcome	Alternative outcome definition				Placebo test	
	Admission excluding observation stays^a^		Admission within 1 day, excluding observation stays^b^		First pre-GED ED visit as pseudo-GED care^c^	
	OR (95% CI)	Absolute difference^d^, percentage points (95% CI)	OR (95% CI)	Absolute difference^d^, percentage points (95% CI)	OR (95% CI)	Absolute difference^d^, percentage points (95% CI)
Hospital admission^e^	0.550**(0.387–0.781)	−11.85**(−18.76 to −4.95)	0.590**(0.418–0.834)	−10.75**(−17.78 to −3.73)	1.006(0.730–1.387)	0.13(−6.36 to 6.61)
No. of persons	4520	4520	4520	4520	4465	4465
30-day mortality	NA NA	NA NA	NA NA	NA NA	0.803(0.414–1.556)	−1.08(−4.34 to 2.18)
No. of persons	NA	NA	NA	NA	4476	4476

*Note:* Significance levels:

** *p* < 0.01.

Abbreviations: ED, Emergency Department; GED, Geriatric Emergency Department.

a Column 1 excludes observation stays from hospital admissions.

b Column 2 defines admission as occurring within 1 day of the ED visit, excluding observation stays.

c Column 3 presents results from a placebo test in which ED visits at sites that later became GEDs but occurred before being classified as GEDs were considered pseudo-GED visits, and visits to sites that never became GEDs during the study period served as controls. The main patient-level analysis was then repeated using the first pseudo-GED visit for patients who had one and the first ED visit for patients who never had a GED visit.

d Absolute differences were estimated from logistic regressions generating predictive margins and represent the change in predicted outcome probabilities between patients who received GED care and those who received non-GED care, adjusted for covariates.

e Patients who died in the outpatient ED before being admitted to an inpatient or observation stay were not at risk for admission and were therefore excluded from the hospital admission outcome.

## Summary

- Key points
  - Receipt of acute care in a Geriatric Emergency Department (GED) was significantly associated with lower odds of hospital admission and 30-day mortality following an emergency department visit.
  - Associations of GED care with hospital admission and mortality were observed among non-Hispanic White patients, and an association with hospital admission was observed among adults younger than 80 years.
  - Despite rapid growth in accredited GED sites, the proportion of older adults receiving care in these settings remains small.
- Why does this paper matter?
  - This is the first nationally representative study to examine hospital admission and mortality associated with GED care after an emergency department visit in older US adults.
  - It highlights the relevance of GED care to acute care outcomes for older adults and underscores the importance of expanding its reach across diverse populations.
