# Supplementary material for: Association of Geriatric Emergency Department Care With Hospitalization and Mortality in Older Adults
Source: J Am Geriatr Soc. Author manuscript; Available in PMC 2026 Aug 1. (PMC13428643; doi:10.1111/jgs.70421)
Supplement: Supplementary Tables [file NIHMS2196537-supplement-Supplementary_Tables.pdf]

Supplementary Materials

Association of Geriatric Emergency Department Care With Hospitalization and Mortality in Older Adults

Supplementary Methods S1: Fuzzy Matching Procedure for Data Linkage

Supplementary Table S1: Weighted Characteristics of Health and Retirement Study Participants Receiving Care in Geriatric Emergency Departments (GEDs) vs Non-GEDs

Supplementary Table S2: Unadjusted Outcomes Among Health and Retirement Study Participants Receiving Care in Geriatric Emergency Departments (GEDs) vs Non-GEDs

Supplementary Table S3: Heterogeneity of the Association Between Receipt of Geriatric Emergency Department Care and Outcomes by Age and Race/Ethnicity

Supplementary Table S4: Sensitivity Analysis Excluding Patients Transferred To or From Another ED

Supplementary Table S5: Sensitivity Analysis Adjusting for Freestanding Emergency Departments

Supplementary Table S6: Association of Geriatric Emergency Department Care With Patient Outcomes Before and During the COVID-19 Pandemic

Supplementary Table S7: Adjusted Association Between Receipt of Geriatric Emergency Department Care and 7-Day Mortality Following an Emergency Department Visit

Supplementary Table S8: Poisson Regression Estimates for the Association Between Receipt of Geriatric Emergency Department Care and Patient Outcomes Following an Emergency Department Visit

Supplementary References

Supplementary Methods S1: Fuzzy Matching Procedure for Data Linkage

To identify emergency departments (EDs) that implemented geriatric emergency department (GED) practices, we relied on the American College of Emergency Physicians (ACEP) accreditation list, which included ED site names and the application initiation date for GED accreditation. Because numeric identifiers were not available, the ACEP list was linked to the American Hospital Association (AHA) database by fuzzy-matching of facility and city names, a string similarity-based method commonly used in prior literature.[1-3]

Fuzzy matching was performed in R using the *fuzzyjoin* package. Facilities were matched between the ACEP and AHA datasets based on facility and city names, restricting matches to the same state. Sites with similarity scores from 0 to 0.114 (n = 269) were classified as exact matches, whereas those with higher scores (n = 232) were manually reviewed by three independent verifiers. Of the 501 domestic EDs listed in the ACEP dataset, 79 had no AHA identifier, and 140 had no corresponding CMS Certification Number (CCN). These 140 EDs were either predominantly government, federally operated facilities for which CCNs are not available (n=61) or had missing CCN identifiers due to missing AHA identifiers in the data (n=79), resulting in 361 EDs with a CCN identifier. Among these 361 EDs, 217 applied for GEDA by the end of 2021, whereas the remaining sites applied after 2021.

The Health and Retirement Study (HRS)-Medicare claims data were linked to the ACEP accreditation list using CCNs to determine GED status and to AHA data using CCNs to incorporate hospital-level characteristics for patients who received GED and non-GED care.

Supplementary Table S1: Weighted Characteristics of Health and Retirement Study Participants Receiving Care in Geriatric Emergency Departments (GEDs) vs Non-GEDs

	No. (%)			
**Characteristics**	**Care in GED[a]**	**Care in Non-GED[a]**	**Full sample**	**P-value**
Number of patients, weighted[b]	1,574,617 (6.2)	23,742,827 (93.8)	25,317,444	
**Patient characteristics**				
Age, y.o.				
65-69	302,690 (19.2)	3,990,274 (16.8)	4,292,964 (17.0)	0.290
70-74	392,385 (24.9)	4,692,971 (19.8)	5,085,356 (20.1)	
75-79	215,418 (13.7)	4,911,599 (20.7)	5,127,017 (20.3)	
80-84	270,589 (17.2)	3,900,626 (16.4)	4,171,215 (16.5)	
≥85	393,535 (25.0)	6,247,357 (26.3)	6,640,892 (26.2)	
Male	687,217 (43.6)	10,087,496 (42.5)	10,774,713 (42.6)	0.781
Race and ethnicity				
Non-Hispanic White	1,212,839 (77.0)	18,851,305 (79.4)	20,064,144 (79.3)	0.261
Non-Hispanic Black	182,350 (11.6)	2,285,054 (9.6)	2,467,404 (9.7)	
Hispanic	111,238 (7.1)	2,016,537 (8.5)	2,127,775 (8.4)	
Non-Hispanic Other	68,190 (4.3)	589,931 (2.5)	658,121 (2.6)	
High school graduate or higher	1,266,912 (80.5)	18,617,284 (78.4)	19,884,196 (78.5)	0.490
With partner	744,736 (47.3)	12,232,499 (51.5)	12,977,235 (51.3)	0.304
Dual-eligible	294,600 (18.7)	4,790,185 (20.2)	5,084,785 (20.1)	0.642
Number of chronic conditions[c], mean (SD)	2.974 (1.481)	3.053 (1.595)	3.048 (1.588)	0.541
ED visit severity				
Emergent	392,588 (24.9)	5,316,610 (22.4)	5,709,198 (22.6)	0.443
Nonemergent	271,566 (17.2)	5,060,657 (21.3)	5,332,223 (21.1)	

Others[d]	910,463 (57.8)	13,365,560 (56.3)	14,276,023 (56.4)	
**Hospital-level characteristics[e]**				
Rural hospital	103,674 (6.6)	3,802,870 (16.0)	3,906,544 (15.4)	<0.001
Teaching hospital				
Major teaching hospital	648,023 (41.2)	2,764,370 (11.6)	3,412,393 (13.5)	<0.001
Minor teaching hospital	668,655 (42.5)	14,371,139 (60.5)	15,039,794 (59.4)	
Non-teaching hospital	257,939 (16.4)	6,607,318 (27.8)	6,865,257 (27.1)	
Total facility beds set up and staffed				
1 to 99	179,099 (11.4)	4,008,414 (16.9)	4,187,513 (16.5)	<0.001
100 to 299	347,251 (22.1)	8,673,378 (36.5)	9,020,629 (35.6)	
300 to 499	510,281 (32.4)	5,721,578 (24.1)	6,231,859 (24.6)	
≥500	537,986 (34.2)	5,339,457 (22.5)	5,877,443 (23.2)	

Abbreviations: ED, emergency department; GED, Geriatric Emergency Department.

[a] The table compares characteristics of patients who received care in GEDs with those who only received care in non-GEDs. The sample included individuals with an ED visit between 2018 and 2020 and was constructed at the patient level using the most recent GED visit for patients who received GED care and the most recent ED visit for those who only received non-GED care.

[b] Weighted characteristics were calculated using Health and Retirement Study (HRS) analytic weights to represent US adults aged 65 years or older.

[c] Chronic conditions data were obtained from the Chronic Condition Warehouse (CCW) linked to the Health and Retirement Study (HRS) survey respondents. Conditions were categorized in alignment with the HRS survey and included eight categories: high blood pressure, diabetes, cancer, lung disease, heart disease, stroke, arthritis, and psychiatric problems. HRS self-reported conditions were used to supplement CCW indicators when CCW data were unavailable. Missing self-reported conditions for a given year were imputed using the most recent non-missing value within the prior 4-year window.

[d] The remaining category includes visits related to injury, mental health, alcohol or drug use, as well as visits that could not be classified.

[e] Hospital-level characteristics were derived from American Hospital Association data linked to Medicare claims and reflect the hospital of each patient's most recent ED visit (the most recent GED visit for GED patients and the most recent ED visit for non-GED patients).

Supplementary Table S2: Unadjusted Outcomes Among Health and Retirement Study Participants Receiving Care in Geriatric Emergency Departments (GEDs) vs Non-GEDs

	No. (%)			
**Outcomes**	**Care in GED[a]**	**Care in Non-GED[a]**	**Total**	**P-value**
**Hospital admission[b]**				
Not admitted following ED visit	99 (36.7)	1,465 (34.5)	1,564 (34.6)	0.462
Admitted following ED visit	171 (63.3)	2,785 (65.5)	2,956 (65.4)	
**Death within 30 days of ED visit**				
Alive within 30 days	233 (86.3)	3,500 (81.5)	3,733 (81.8)	0.049
Died within 30 days	37 (13.7)	793 (18.5)	830 (18.2)	

Abbreviations: ED, emergency department; GED, Geriatric Emergency Department.
[a] The table presents unadjusted outcomes (hospital admission and 30-day mortality) for patients receiving care in GEDs vs non-GEDs. Outcomes were assessed using each patient's most recent ED visit (the most recent GED visit for patients seen in a GED and the most recent ED visit for those never seen in a GED).
[b] Patients who died in the outpatient ED before being admitted to an inpatient or observation stay were not at risk for admission and were therefore excluded from the hospital admission outcome.

Supplementary Table S3: Heterogeneity of the Association Between Receipt of Geriatric Emergency Department Care and Outcomes by Age and Race/Ethnicity

	Hospital admission	Death within 30 days of ED visit
**Variables**	**OR (95% CI)**	**OR (95% CI)**
Panel A. Logistic regression with Age × GED care interaction		
GED × Age ≥ 80	2.182* (1.102 - 4.320)	1.016 (0.412 - 2.506)
GED	0.442** (0.259 - 0.757)	0.622 (0.296 - 1.304)
Age ≥ 80	1.116 (0.835 - 1.491)	1.117 (0.799 – 1.560)
P value for interaction	0.025	0.972
Observations	4,520	4,563
Panel B. Logistic regression with race/ethnicity × GED care interaction		
GED × Non-Hispanic White	0.377* (0.177 - 0.803)	0.540 (0.207 - 1.405)
GED	1.330 (0.708 - 2.498)	0.951 (0.430 – 2.105)
Non-Hispanic White	0.924 (0.743 - 1.151)	1.090 (0.840 - 1.415)
P value for interaction	0.012	0.206
Observations	4,520	4,563

Abbreviations: ED, emergency department; GED, Geriatric Emergency Department.
Notes: The table presents estimates from logistic regression models in which the outcome is regressed on receipt of GED care, each subgroup indicator, and their interaction terms, adjusting for the same covariates as in the main analysis. The statistical significance of the interaction term indicates whether the association between GED care and the outcome differs across subgroups.
Significance levels: ** $p < 0.01$, * $p < 0.05$.

Supplementary Table S4: Sensitivity Analysis Excluding Patients Transferred To or From Another ED

Samples	Hospital admission		Death within 30 days of ED visit	
	OR (95% CI)	Absolute Difference[a], percentage points (95% CI)	OR (95% CI)	Absolute Difference[a], percentage points (95% CI)
Excluded Patients Transferred To or From Another ED[b]	0.578**	-10.68**	0.596*	-6.34*
	(0.397 to 0.840)	(-17.93 to -3.43)	(0.382 to 0.930)	(-11.83 to -0.85)
Observations	4,334	4,334	4,375	4,375

Abbreviations: ED, emergency department.
[a] Absolute differences were estimated from logistic regressions generating predictive margins and represent the change in predicted outcome probabilities between patients who received GED care and those who received non-GED care, adjusted for covariates.
[b] We identified potential ED-to-ED transfers using discharge status and visit timing across consecutive ED claims for the same patient. Specifically, a visit was classified as a transfer-in if the prior claim's discharge status indicated transfer/discharge to a short-term hospital, federal hospital, or critical access hospital, and if the gap between the prior discharge date and the current ED visit date was ≤1 day. A visit was classified as a transfer-out if the discharge status on the ED visit indicated transfer to one of these facility types.
Significance levels: ** $p < 0.01$, * $p < 0.05$.

Supplementary Table S5: Sensitivity Analysis Adjusting for Freestanding Emergency Departments

	Hospital admission		Death within 30 days of ED visit	
Samples	OR (95% CI)	Absolute Difference[a], percentage points (95% CI)	OR (95% CI)	Absolute Difference[a], percentage points (95% CI)
All	0.568**	-10.95**	0.554*	-7.76*
	(0.383 to 0.843)	(-18.56 to -3.34)	(0.347 to 0.884)	(-13.94 to -1.58)
Observations[b]	3,740	3,740	3,775	3,775

Abbreviations: ED, emergency department.

[a] Absolute differences were estimated from logistic regressions generating predictive margins and represent the change in predicted outcome probabilities between patients who received GED care and those who received non-GED care, adjusted for covariates.

[b] This sensitivity analysis additionally adjusted for hospital-owned freestanding emergency departments (FSEDs), identified using the off-campus emergency department indicator in the American Hospital Association (AHA) data. Because this variable has 18% missingness, the sample size is smaller than in the main analysis.

Significance levels: ** $p < 0.01$, * $p < 0.05$.

Supplementary Table S6: Association of Geriatric Emergency Department Care With Patient Outcomes Before and During the COVID-19 Pandemic

	Hospital admission		Death within 30 days of ED visit	
**Samples**	**OR (95% CI)**	**Absolute Difference[a], percentage points (95% CI)**	**OR (95% CI)**	**Absolute Difference[a], percentage points (95% CI)**
Panel A. Pre-COVID-19[b]				
All	0.444**	-15.94**	0.390*	-9.06*
	(0.254 - 0.777)	(-26.86 to -5.02)	(0.153 – 0.992)	(-18.08 to -0.05)
Observations	3,265	3,265	3,290	3,290
Panel B. During-COVID-19[b]				
All	0.724	-6.44	0.780	-2.85
	(0.483 - 1.084)	(-14.48 to 1.60)	(0.476 - 1.276)	(-8.51 to 2.81)
Observations	2,585	2,585	2,603	2,603

[a] Absolute differences were estimated from logistic regressions generating predictive margins and represent the change in predicted outcome probabilities between patients who received GED care and those who received non-GED care, adjusted for covariates.
[b] The pre-COVID-19 period was defined as up to February 2020, and the during-COVID-19 period as March 2020 onward. For each period, we used each patient's most recent ED visit (the most recent GED visit for those receiving GED care and the most recent ED visit for those receiving non-GED care) and repeated the main adjusted analysis.
Significance levels: ** $p < 0.01$, * $p < 0.05$.

Supplementary Table S7: Adjusted Association Between Receipt of Geriatric Emergency Department Care and 7-Day Mortality Following an Emergency Department Visit

	Death within 7 days of ED visit	
**Samples**	**OR (95% CI)**	**Absolute Difference[a], percentage points (95% CI)**
All	0.726	-1.89
	(0.380 to 1.388)	(-5.74 to 1.95)
Observations	4,563	4,563

Abbreviations: ED, emergency department.

[a] Absolute differences were estimated from logistic regressions generating predictive margins and represent the change in predicted outcome probabilities between patients who received GED care and those who received non-GED care, adjusted for covariates.

Supplementary Table S8: Poisson Regression Estimates for the Association Between Receipt of Geriatric Emergency Department Care and Patient Outcomes Following an Emergency Department Visit

	**Hospital admission**[a]	**Death within 30 days of ED visit**
**Samples**	**RR**[b] **(95% CI)**	**RR**[b] **(95% CI)**
Panel A. Overall sample	0.843*	0.677*
	(0.735 - 0.966)	(0.473 - 0.970)
Observations	4,520	4,563
Panel B. Subgroups by age		
Age ≥ 80 y.o.	0.986	0.712
	(0.882 - 1.103)	(0.480 - 1.056)
Observations	2,400	2,425
Age < 80 y.o.	0.721*	0.639
	(0.558 - 0.932)	(0.325 - 1.259)
Observations	2,120	2,138
Panel C. Subgroups by race/ethnicity		
White[c]	0.781**	0.573*
	(0.649 - 0.939)	(0.361 - 0.910)
Observations	3,104	3,126
Non-white[c]	1.037	0.996
	(0.904 - 1.190)	(0.564 - 1.759)
Observations	1,416	1,437

Abbreviations: ED, emergency department.
[a] Patients who died in the outpatient ED before being admitted to an inpatient or observation stay were not at risk for admission and were therefore excluded from the hospital admission outcome.
[b] Risk ratios (RR) were estimated using Poisson regression with robust error variances.
[c] White subgroups refer to non-Hispanic White individuals, and non-White subgroups refer to other racial and ethnic groups, including non-Hispanic Black, Hispanic, and others.
Significance levels: ** $p < 0.01$, * $p < 0.05$.

Supplementary References

1. Bruch JD, Foot C, Singh Y, Song ZR, Polsky D, Zhu JM. Workforce Composition In Private Equity-Acquired Versus Non- Private Equity-Acquired Physician Practices. *Health Affair*. Jan 2023;42(1):121-129. doi:10.1377/hlthaff.2022.00308

2. "Health System Ownership Of Medicare Advantage Plans Has Increased Over Time", Health Affairs Forefront, July 18, 2025. DOI: 10.1377/forefront.20250717.786759.

3. Chen Z, Wang Y, Narasayya V, Chaudhuri S. Customizable and scalable fuzzy join for big data. *Proceedings of the VLDB Endowment*. 2019;12(12):2106-2117.
